# The effects of unexpected mechanical perturbations during treadmill walking on spatiotemporal gait parameters, and the dynamic stability measures by which to quantify postural response

**DOI:** 10.1371/journal.pone.0195902

**Published:** 2018-04-19

**Authors:** Forough Madehkhaksar, Jochen Klenk, Kim Sczuka, Katharina Gordt, Itshak Melzer, Michael Schwenk

**Affiliations:** 1 Department of Sports Sciences, Heidelberg University, Heidelberg, Germany; 2 Department of Clinical Gerontology and Rehabilitation, Robert-Bosch-Hospital, Stuttgart, Germany; 3 Insitute of Epidemiology and Medical Biometry, Ulm University, Ulm, Germany; 4 Network Aging Research (NAR), Heidelberg University, Heidelberg, Germany; 5 Department of Physical Therapy, Faculty of Health Sciences, Ben-Gurion University of the Negev, Beer-Sheva, Israel; Purdue University, UNITED STATES

## Abstract

Most falls occur after a loss of balance following an unexpected perturbation such as a slip or a trip. Greater understanding of how humans control and maintain stability during perturbed walking may help to develop appropriate fall prevention programs. The aim of this study was to examine changes in spatiotemporal gait and stability parameters in response to sudden mechanical perturbations in medio-lateral (ML) and anterior-posterior (AP) direction during treadmill walking. Moreover, we aimed to evaluate which parameters are most representative to quantify postural recovery responses. Ten healthy adults (mean = 26.4, SD = 4.1 years) walked on a treadmill that provided unexpected discrete ML and AP surface horizontal perturbations. Participants walked under no perturbation (normal walking), and under left, right, forward, and backward sudden mechanical perturbation conditions. Gait parameters were computed including stride length (SL), step width (SW), and cadence, as well as dynamic stability in AP- (MoS-AP) and ML- (MoS-ML) directions. Gait and stability parameters were quantified by means, variability, and extreme values. Overall, participants walked with a shorter stride length, a wider step width, and a higher cadence during perturbed walking, but despite this, the effect of perturbations on means of SW and MoS-ML was not statistically significant. These effects were found to be significantly greater when the perturbations were applied toward the ML-direction. Variabilities, as well as extremes of gait-related parameters, showed strong responses to the perturbations. The higher variability as a response to perturbations might be an indicator of instability and fall risk, on the same note, an adaptation strategy and beneficial to recover balance. Parameters identified in this study may represent useful indicators of locomotor adaptation to successfully compensate sudden mechanical perturbation during walking. The potential association of the extracted parameters with fall risk needs to be determined in fall-prone populations.

## Introduction

Falls are a serious clinical problem and often lead to injuries, the decline in mobility, and self-imposed limitations on daily activities, especially in older adults. Fall-related injuries increase costs for health care and rehabilitation and diminish the quality of life [1–3]. Most falls occur after a loss of balance while walking, which is the most common activity in daily life, and following an unexpected perturbation such as a slip or trip [4]. Therefore, understanding of how humans control balance and maintain stability during unexpected perturbed walking can help with assessment of balance recovery ability and thus may help to reduce the incidence of falls.

In order to enhance understanding of falls caused by perturbations, recent studies have examined changes in spatiotemporal gait parameters and dynamic stability (i.e., the margins of stability [5,6]) following perturbations. Evidence has demonstrated adaptations of spatiotemporal gait parameters to challenged walking by taking faster, shorter, and wider steps [7–11]. Consequently, an alteration in gait parameters led to increased margins of stability (MoS) and to enhanced stability during challenging walking [8,9]. While these alterations in spatiotemporal gait parameters and dynamic stability occurred during different types of perturbations, such as continuous mechanical and visual perturbations [9–14], it remains inconclusive whether these observable adaptations also occur during sudden mechanical surface perturbations in different directions.

The majority of perturbation studies has included perturbations only in the anterior-posterior (AP) [7,15–17] or in the medio-lateral (ML) direction [9,11,13,14,18,19]. However, each of these perturbations affects gait and stability in different ways, depending not only on the type but also on the direction of the perturbations. Exposure to the continuous support surface [10,12] and visual field [10,20,21] in both AP- and ML-directions produced anisotropic changes in gait variabilities. The effects of perturbations were also found to be significantly greater when perturbations were applied in the ML-direction [10,12,21]. Also, the unidirectionality (AP or ML) of the perturbation may help the subjects in developing a volitional plan for a stepping response thus lack’s the ecological validity since falls in the real world are multidirectional and always unexpected [22,23]. Therefore, further studies on the effect of perturbations on gait-related parameters and dynamic stability, which include sudden mechanical surface perturbation in both AP- and ML-directions may reveal valuable information.

The means of gait characteristic appeared resistant to the effect of challenging walking depending on the challenge [18,24]. Alternatively, the response of variability to perturbations was stronger than the response of means during the continuous platform and visual perturbations [12]. This indicated an increased challenge in stability that was not captured by means but by the variability of parameters [12]. Thus, gait variability, which is defined as fluctuation in gait parameters from one step to the next, might be an important indicator of gait stability [25,26], and more responsive than the mean differences of the gait parameters.

Prior studies have used gait variability to characterize balance during walking [10,11,18,21,27]. However, studies on the response of variability of the gait parameters to perturbations provided contradictory results. Continuous support surface perturbations during walking in a static visual environment induced increased step width variability [14]. On the other hand, Francis et al. reported no significant increase in gait variability in young adults in response to visual ML perturbation [18]. These differences might appear due to different types of perturbations applied in these studies. In a recent work, Punt et al. explored the effects of multidirectional sudden mechanical perturbations in stroke survivors who prospectively experienced falls or no falls [28]. By comparing the gait characteristics and dynamic stability in both fallers and non-fallers group over every step after the perturbation, they observed no difference in individual’s ability to cope with the perturbations. Although their study provided interesting insight into the response strategy in stroke survivors, the variability of the parameters which might reveal helpful information in discriminations between fallers and non-fallers was not included. There is a need for studies which examine the effect of sudden multidirectional unexpected mechanical perturbations on the variability of gait-related parameters.

Additionally, extremes of gait-related parameters may be a better representative estimate of the parameters in a challenging condition, such as perturbed walking compared with the mean values that traditionally being used in research [29]. Rispens et al. found a strong association between extremes relating to high gait quality and fall risk during daily life walking. During perturbed treadmill walking, extremes may better capture pronounced postural responses after perturbations, and in turn may be more sensitive indicators of gait stability [29]. To the best of our knowledge, there have been no studies to evaluate the response of extremes of gait-related parameters to quantify postural stability during perturbed walking.

The first aim of this study was to examine the changes in a candidate set of spatiotemporal gait and stability parameters in response to sudden unexpected multidirectional mechanical perturbations. Secondly, we aimed to evaluate the most affected parameters of this set for measuring the effect of perturbations on postural recovery responses. Means, variability, and extremes of gait-related parameters were used to specify responses during perturbed treadmill walking. We hypothesized that participants would exhibit: (1) alterations in spatiotemporal gait parameters to enhance dynamic stability and (2) a greater effect of perturbations on extremes and variability of measures, as compared to means.

## Methods

### Participants and experimental protocol

Ten healthy young adults (age: 26.4 ± 4.1 years, height: 1.7 ± 0.08 m, mass: 64.4 ± 12.5 kg, 7 females) participated in this study. All participants provided written informed consent and the study was approved by the ethical committee of the Medical Faculty, Tübingen University. Recruited subjects had no experience of walking on the perturbation treadmill.

**Fig 1:**
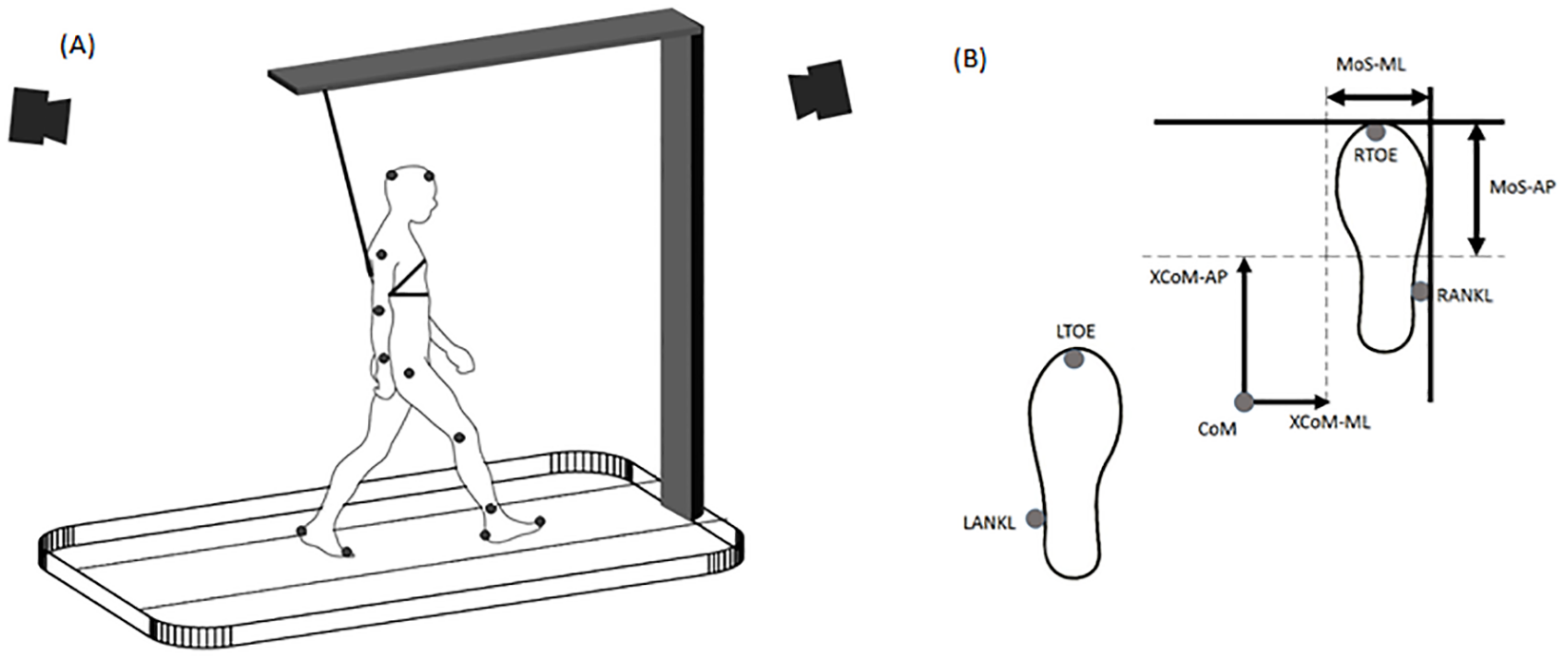
(A) A schematic drawing of the experimental setup. Forward and backward perturbations were induced by acceleration and deceleration of the treadmill’s belt. Left and right perturbations were induced by moving the treadmill surface in the ML-direction. Reflective markers were placed at specific anatomical locations in accordance with the plug-in-gait marker set. (B) MoS-AP was defined as the AP distance between the XCoM-AP and the anterior boundary of the BoS, defined by the leading toe marker (either RTOE or LTOE for the right and the left foot, respectively). MoS-ML was defined as the ML distance between the XCoM-ML and the lateral boundary of the BoS, defined by the ankle marker (RANKL and LANKL for the right and the left foot, respectively).

Participants walked on a perturbation treadmill (Balance Tutor, MediTouch, Netanya, Israel) at the fixed speed of 1.11 ms^-1^ and were subjected to unexpected surface perturbations in left, right, forward, and backward directions (Fig 1). The system has been described in detail previously [30]. The treadmill platform is mounted on linear slides, which allow to translate it in the lateral direction. Left and right perturbations were induced by automatically moving the treadmill surface in ML-direction (12.8 cm and 1.5 ms^-2^). Forward and backward perturbations were induced by acceleration and deceleration of the belt. To present the forward perturbation, the belt speed accelerated toward 2.5 ms^-1^ and subsequently decelerated toward 1.1 ms^-1^. The backward perturbation was presented by deceleration of the belt speed toward 0 ms^-1^ and subsequent acceleration toward 1.1 ms^-1^. First, the subjects completed 5 minutes (min) of normal walking on the perturbation treadmill without perturbations to become familiar with treadmill walking. The last min of the treadmill walking trial was used for data analysis (Normal) in order to measure the subject’s normal walking pattern. Afterwards, 4 trials of 1 min perturbation treadmill walking were recorded. During each trial, participants were exposed to a single perturbation in a specific direction in order to become familiar with perturbed walking. Subsequently, 4 trials of 5 min perturbation treadmill walking including a series of 16 perturbations towards a specific direction were recorded. The moment of all perturbations was unpredictable. The time interval between perturbations ranged from 15–25 sec. All participants walked in their comfortable sport shoes. Subjects always wore a loss safety harness to prevent falls that prevented falls but did not restrict their gait.

### Measurements and data analysis

Kinematic data were recorded at 200 Hz with an eight cameras motion capture system (Vicon Motion System, Oxford, UK). A total of 39 reflective markers were placed at specific anatomical locations in accordance with the Plug-In-Gait marker set (Bodybuilder, Plug in Gait model, Vicon Motion Systems, Oxford, UK). Motion data was analyzed using the Vicon Nexus software (Version 2.5). The time frame of interest was 15 sec including 5 sec before and 10 sec after the perturbation.

Spatiotemporal gait parameters including step length, step width, and cadence were measured at the instant of the heel strike. Heel strike was identified as the local maxima of the position of the heel markers in the AP-direction [31]. Stride length was defined as the AP-distance between heel markers at the instant of heel strike plus the treadmill translation during the stride. Step width was measured as the ML-distance between ankle markers at the moment of heel strike. Cadence was calculated as the number of steps per minute.

Dynamic margins of stability were adapted from Hof et al. [5]. In this study, the extrapolated center of mass (XCoM) was calculated as the position of the center of mass (CoM), plus its velocity multiplied by the factor $\sqrt{lg^{-1}}$, where g was the acceleration of gravity and l was the distance from the ankle marker of the trailing foot to the CoM at the instant of heel strike. The margins of stability in the anterior-posterior direction (MoS-AP) were calculated as the AP distance between the XCoM and the toe marker of the leading foot. The margins of stability in the ML-direction (MoS-ML) were calculated as the lateral distance between the XCoM and the ankle marker of the leading foot (Fig 1). MoS was calculated at heel strike for every step during each time frame (~ 24 steps per each 15 sec time frame). All processing and analyses were performed with custom MATLAB R2015a programs (Mathworks, Inc., Natic, USA). Measured values were visually checked regarding plausibility and wrong values resulted from an error in the calculations due to the disturbed trajectory of markers were removed for further analyzing.

For each time frame of 15 sec treadmill walking, the mean from all steps performed was calculated for each spatiotemporal gait parameter and MoS. Additionally, variability characterized as the standard deviation was calculated for each spatiotemporal gait parameter and MoS. Thus, gait characteristics were measured as the mean (mn) and standard deviation (sd) of the spatiotemporal gait parameters including stride length (SL_mn_ and SL_sd_), step width (SW_mn_ and SW_sd_), and cadence (cadence_mn_ and cadence_sd_). Dynamic stability was calculated as the mean and standard deviation of MoS in AP- (MoS-AP_mn_ and MoS-AP_sd_) and ML- (MoS-ML_mn_ and MoS-ML_sd_) directions.

In addition, extremes were estimated as the 10th and 90th percentiles of the stride length (SL_P10_ and SL_P90_), step width (SW_P10_ and SW_P90_), and cadence (cadence _P10_ and cadence _P90_), as well as MoS in AP- (MoS-AP_P10_ and MoS-AP_P90_) and ML- (MoS-ML_P10_ and MoS-ML_P90_) directions.

### Statistical analysis

Multiple measures of variable including the mean, variability, and extremes of the spatiotemporal gait parameters as well as MoS in ML- and AP-directions were reduced to the mean values for each walking condition. Paired t-test and corresponding confidence interval (CI) was used to examine differences between normal walking and perturbed walking conditions. In addition, the effect size of responses was calculated using Cohen’s *d* statistic (*d*) to describe the strength of the effect of perturbation conditions on each measurement. Cohen’s *d* statistic was defined as the mean difference between normal and perturbed walking conditions divided by the standard deviation of changes between conditions.

All statistical analyses were performed using SAS software, version 9.4 (SAS Institute Inc., Cary, NC, USA) with a confidence interval of 95% for all comparisons.

## Results

**Fig 2:**
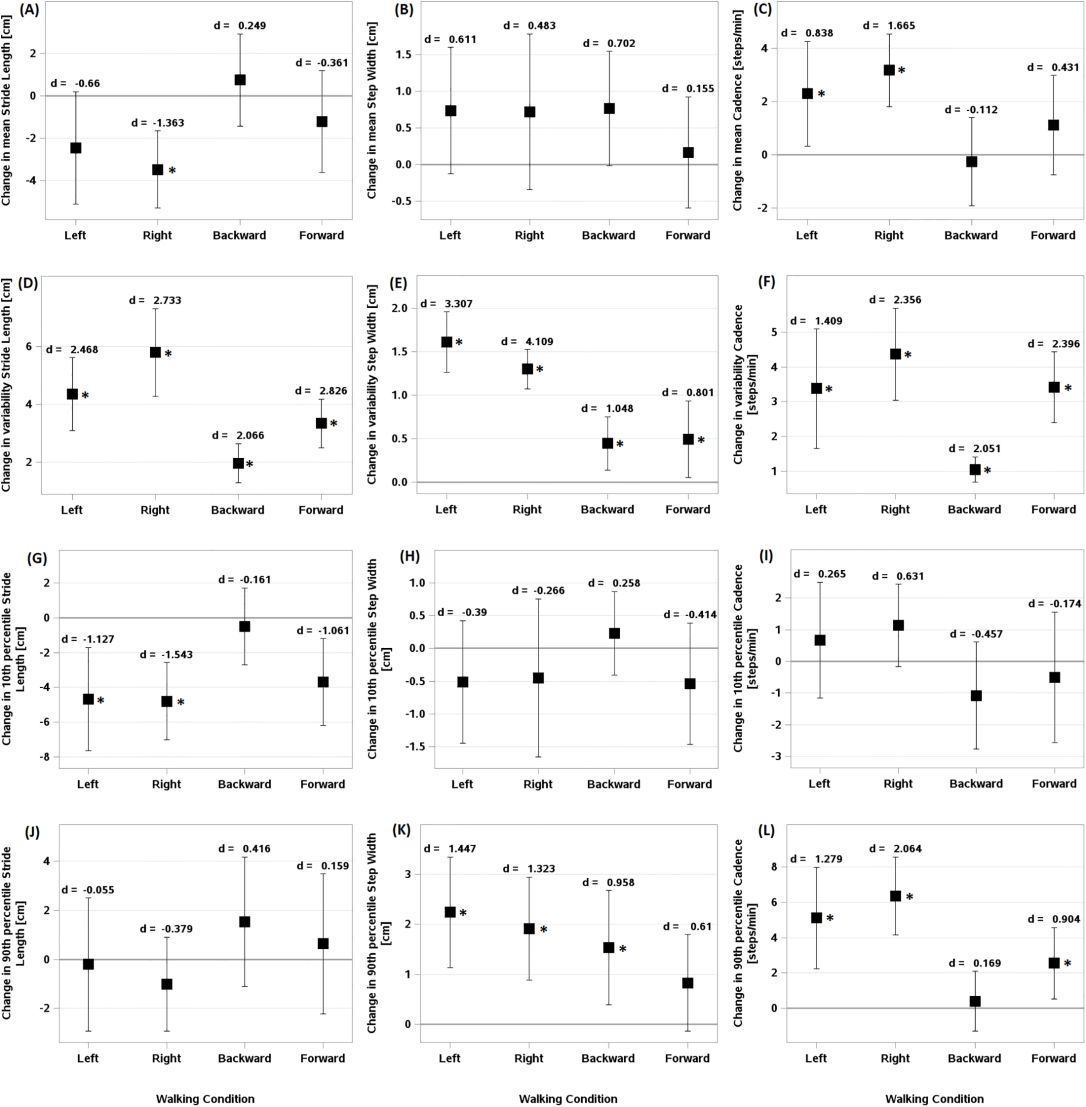
Difference of means, variability, and extremes of spatiotemporal gait parameters during perturbed walking conditions relative to normal walking. Difference of means of (A) stride length, (B) step width, and (C) cadence. Difference of variability of (D) stride length, (E) step width, and (F) cadence. Difference of 10th percentile of (G) stride length, (H) step width, and (I) cadence. Difference of 90th percentile of (J) stride length, (K) step width, and (L) cadence. *d* indicates Cohen’s *d* statistic effect size. Error bars indicate confidence intervals. (*) indicates statistically significant differences from Normal walking.

**Fig 3:**
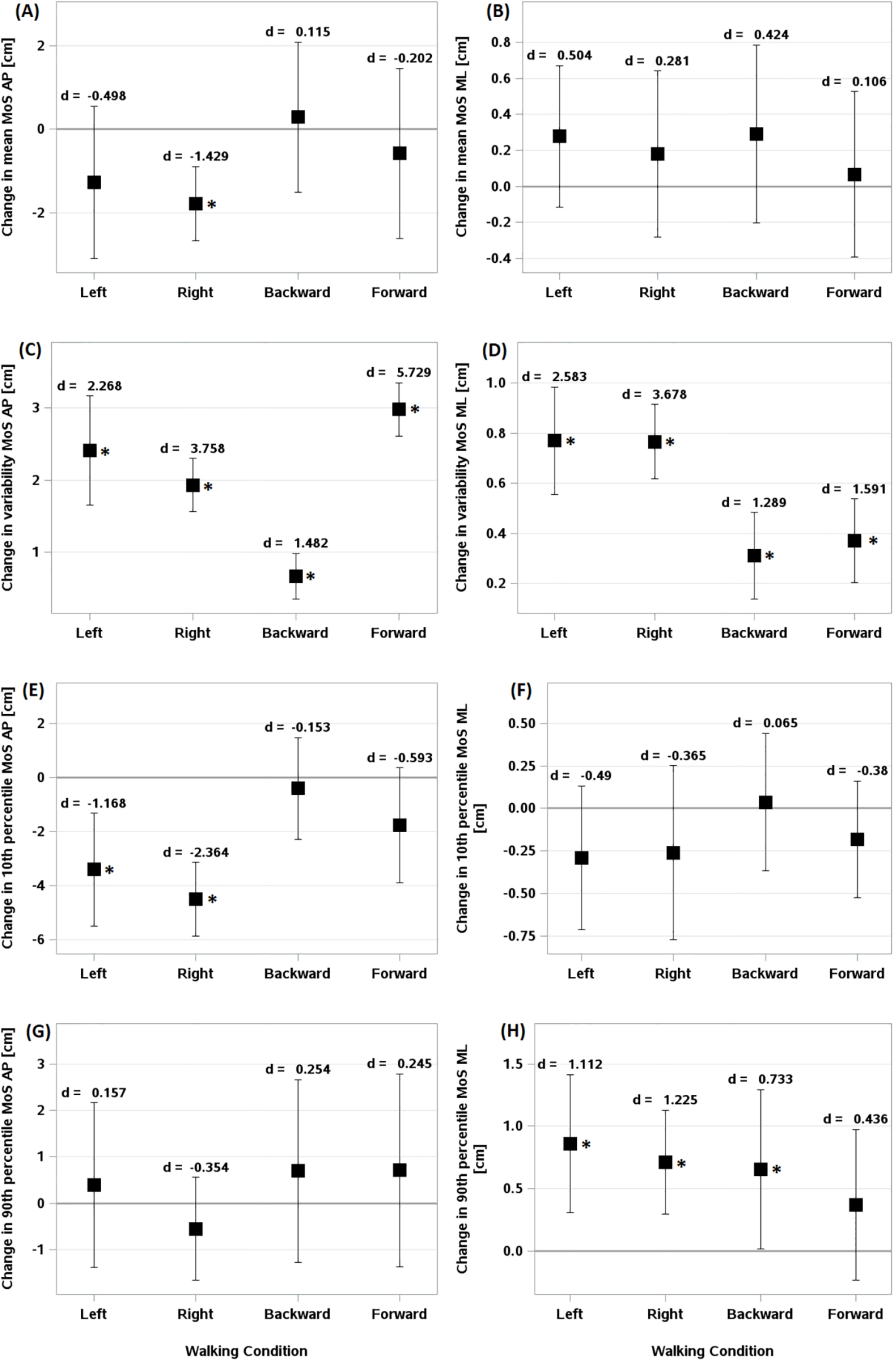
Difference of means, variability, and extremes of dynamic stability during perturbed walking conditions relative to normal walking. Difference of means of (A) MoS-AP and (B) MoS-ML. difference of variability of (C) MoS-AP and (D) MoS-ML. difference of 10th percentile of (E) MoS-AP and (F) MoS-ML. difference of 90th percentile of (G) MoS-AP and (H) MoS-ML. *d* indicates Cohen’s *d* statistic effect size. Error bars indicate confidence intervals. (*) indicates statistically significant differences from Normal walking.

All subjects completed the experiment with no fall into the harness system during the perturbation trials. In total, 116 left, 130 right, 141 forward, and 144 backward perturbations were analyzed. The results for means, variabilities, and extremes of normal walking, as well as perturbed walking, are presented in Table 1. Also, results of statistical analyses including mean differences of perturbed walking conditions relative to normal walking, as well as the associated CI and effect sizes (i.e., Cohen’s *d* statistic) are presented in Figs 2 and 3.

**Table 1:** Results for spatiotemporal gait parameters and margins of stability during different walking conditions (mean and SD; n = 10).

	Condition				
	Normal	Left	Right	Backward	Forward
Stride length [cm]					
Mean	128.83 ± 8.68	126.38 ± 7.56	125.35 ± 7.98	129.59 ± 7.54	127.61 ± 7.56
Variability	2.08 ± 0.48	6.43 ± 1.75	7.86 ± 1.98	4.03 ± 0.78	5.41 ± 1.09
P10	126.32 ± 8.55	121.65 ± 6.61	121.52 ± 7.53	125.82 ± 7.25	122.62 ± 7.76
P90	131.54 ± 8.78	131.34 ± 8.00	130.53 ± 8.40	133.08 ± 8.09	132.18 ± 8.14
Step width [cm]					
Mean	20.97 ± 2.92	21.71 ± 3.30	21.69 ± 3.51	21.74 ± 3.22	21.14 ± 3.32
Variability	1.57 ± 0.39	3.18 ± 0.53	2.87 ± 0.38	2.02 ± 0.60	2.06 ± 0.72
P10	19.06 ± 2.86	18.56 ± 3.29	18.62 ± 3.70	19.29 ± 3.42	18.53 ± 3.46
P90	22.96 ± 3.03	25.19 ± 3.29	24.87 ± 3.34	24.49 ± 3.46	23.78 ± 3.52
Cadence [steps/min]					
Mean	103.96 ± 5.49	106.26 ± 6.26	107.14 ± 6.67	103.70 ± 5.72	105.08 ± 5.94
Variability	1.45 ± 0.40	4.83 ± 2.28	5.81 ± 1.76	2.50 ± 0.44	4.87 ± 1.35
P10	102.14 ± 6.63	102.81 ± 6.27	103.28 ± 6.33	101.06 ± 5.73	101.64 ± 5.89
P90	105.85 ± 6.64	110.96 ± 6.87	112.19 ± 7.09	106.25 ± 5.62	108.39 ± 5.81
MoS-ML [cm]					
Mean	8.89 ± 1.24	9.17 ± 1.41	9.07 ± 1.48	9.19 ± 1.38	8.92 ± 1.51
Variability	0.67 ± 0.16	1.43 ± 0.27	1.42 ± 0.18	0.97 ± 0.24	1.03 ± 0.20
P10	8.01 ± 1.30	7.73 ± 1.25	7.75 ± 1.58	8.05 ± 1.38	7.83 ± 1.39
P90	9.76 ± 1.26	10.62 ± 1.61	10.48 ± 1.46	10.42 ± 1.53	10.13 ± 1.62
MoS-AP [cm]					
Mean	9.38 ± 2.86	8.11 ± 2.39	7.61 ± 2.35	9.67 ± 2.64	8.81 ± 2.66
Variability	0.96 ± 0.25	3.37 ± 1.01	2.89 ± 0.55	1.62 ± 0.51	3.94 ± 0.48
P10	8.17 ± 3.00	4.78 ± 2.55	3.67 ± 2.57	7.78 ± 2.99	6.41 ± 2.41
P90	10.62 ± 2.69	11.01 ± 2.34	10.07 ± 2.17	11.32 ± 2.46	11.33 ± 2.74

### Means of gait parameters and dynamic stability

Overall, compared with unperturbed treadmill walking, participants walked with shorter stride length, wider step width, and higher cadence during ML perturbations. However, the effect of perturbations on SW_mn_ was not statistically significant (Fig 2A, 2B and 2C). Exposure to the right perturbation resulted in a significantly shorter stride length (Est. = -3.478, 95% CI [-5.302, -1.652], d = -1.363). In left perturbation, participants tended to decrease their stride length (Est. = -2.448, 95% CI [-5.101, 0.206], d = -0.66). However, there were no significant differences in SL_mn_, SW_mn_, and Cadence_mn_ during forward and backward perturbations compared with unperturbed walking (Fig 2A, 2B and 2C).

Similar to SL_mn_, exposure to right perturbation resulted in significantly shorter MoS-AP_mn_ compared with unperturbed walking (Est. = -1.776, 95% CI [-2.665, -0.887], d = -1.429, Fig 3A). Also, MoS-AP_mn_ tended to decrease during left perturbation, however, the effect did not reach to the significant level (Est. = -1.269, 95% CI [-3.093, 0.555], d = -0.498). The perturbations had no significant effect on MoS-ML_mn_ (Fig 3B).

### Variability of gait parameters and dynamic stability

During all perturbation conditions, the variability of stride length, step width, and cadence was significantly higher than during unperturbed walking (Fig 2D, 2E and 2F). Lateral perturbations resulted in an increase in the variability of stride length and step width than forward and backward perturbations. However, the strength of the effect on stride length variability appeared high during all perturbation conditions (Left: Est. = 4.352, 95% CI [3.091, 5.613], d = 2.468; Right: Est. = 5.784, 95% CI [4.271, 7.298], d = 2.733; Backward: Est. = 1.955, 95% CI [1.278, 2.632], d = 2.066; Forward: Est. = 3.331, 95% CI [2.488, 4.175], d = 2.826, Fig 2D). On the other hand, the results of SW_sd_ exhibited stronger effect of lateral perturbations than forward and backward perturbations (Left: Est. = 1.609, 95% CI [1.261, 1.958], d = 3.307; Right: Est. = 1.299, 95% CI [1.073, 1.526], d = 4.109; Backward: Est. = 0.448, 95% CI [0.142, 0.754], d = 1.048; Forward: Est. = 0.495, 95% CI [0.053, 0.937], d = 0.801, Fig 2E).

Similar to the results of gait parameters, the dynamic stability exhibited significantly greater variability during all perturbation conditions relative to unperturbed treadmill walking (Fig 3C and 3D). However, forward perturbation had greater effect on MoS-AP_sd_ than on MoS-ML_sd_ (Est. = 2.979, 95% CI [2.607, 3.351], d = 5.729 and Est. = 0.371, 95% CI [0.204, 0.537], d = 1.591, respectively).

### Extreme values

The results for extremes of spatiotemporal gait parameters showed no significant differences between SL_P90_, SW_P10_, and Cadence_P10_ during perturbation walking conditions compared with unperturbed treadmill walking (Fig 2J, 2H and 2I). SL_P10_ during lateral and forward perturbations was significantly shorter than during unperturbed walking (Left: Est. = -4.663, 95% CI [-7.624, -1.702], d = -1.127; Right: Est. = -4.794, 95% CI [-7.017, -2.572], d = -1.543; Forward: Est. = -3.699, 95% CI [-6.192, -1.205], d = -1.061, Fig 2G). Also, SW_P90_ significantly increased during lateral and backward perturbations (Left: Est. = 2.239, 95% CI [1.132, 3.347], d = 1.447; Right: Est. = 1.913, 95% CI [0.879, 2.948], d = 1.323; Backward: Est. = 1.534, 95% CI [0.389, 2.679], d = 0.958, Fig 2K). In addition, cadence_P90_ during sideway and forward perturbations was significantly greater than during unperturbed walking, however the effect of lateral perturbations was stronger compared with backward perturbation (Left: Est. = 5.11, 95% CI [2.253, 7.968], d = 1.279; Right: Est. = 6.349, 95% CI [4.148, 8.549], d = 2.064; Forward: Est. = 2.549, 95% CI [0.531, 4.568], d = 0.904, Fig 2L).

Similar to the results of step width, MoS-ML_P90_ during lateral and backward perturbations was significantly larger than during unperturbed walking (Left: Est. = 0.861, 95% CI [0.307, 1.414], d = 1.112; Right: Est. = 0.714, 95% CI [0.297, 1.131], d = 1.225; Backward: Est. = 0.656, 95% CI [0.016, 1.297], d = 0.733, Fig 3H). However, the results of MoS-ML_P10_ showed no significant change between perturbed and unperturbed gait (Fig 3F). Also, MoS-AP_P90_ was not significantly different between perturbed and unperturbed treadmill walking (Fig 3G), whereas MoS-AP_P10_ during ML perturbation was significantly greater than during unperturbed walking (Left: Est. = -3.401, 95% CI [-5.484, -1.318], d = -1.168; Right: Est. = -4.505, 95% CI [-5.868, -3.142], d = -2.364, Fig 3E).

## Discussion

In this study, we found that spatiotemporal gait parameters, as well as MoS, were affected during exposure to AP- and ML- perturbations depending on the direction of the perturbations. Participants took shorter, wider, and faster steps in order to increase their dynamic stability in balance recovery during walking. More noteworthy was the increase in variability of these parameters relative to unperturbed walking. These effects were also found to be significantly greater when the perturbations were applied in the ML-direction.

Interestingly and as one might have expected by theory, the response of stride length (i.e. AP response of spatial gait parameters) and MoS-AP (i.e. AP response of dynamic stability) exhibited the same pattern of response to perturbations. Similarly, the response pattern of step width (i.e. ML response of spatial gait parameters) and MoS-ML (i.e. ML response of dynamic stability) appeared comparable. In addition, the response pattern of cadence (i.e., temporal gait parameter) was reversely the same as that for stride length. Based on the theoretical models, in which the human body during walking is modeled as a simple inverted pendulum, cadence, stride length, and walking speed cannot be adapted independently from each other [5,6,8,32]. In the present study, subjects walked on the treadmill with a fixed walking speed, therefore cadence was adapted according to the stride length.

Previous studies showed decreases in stride length, increases in step width and cadence with increasing perturbation intensity [9–11,33]. In this study, subjects exhibited shorter, larger, and faster steps during ML than AP perturbations, suggesting that ML perturbations were more challenging than AP perturbations, which is consistent with McIntosh et al. who used ML and AP overground platform perturbations during walking [34]. However, they quantified responses by CoM displacement and velocity, thus it remained unknown to what extent the stability of gait was affected by perturbations.

In line with previous studies, MoS-AP significantly decreased in response to ML perturbations [12]. MoS-AP is defined as the distance between the AP boundaries of the base of support (BoS) and XCoM. Shorter and faster steps, which bring the CoM closer to the moving BoS, improved stability in AP-direction [7,9,32,33]. Conversely, MoS-ML slightly increased in response to applied perturbations implies a decrease in risk of falling [9,12]. Similar to the previous studies, our results show that lateral dynamic stability was controlled by taking slightly wider steps to maintain stable walking during the perturbed walking [6,9,12]. The MoS in ML direction is defined as the distance between the ML borders of the BoS and XCoM. Thus, increased step width resulted in an increase in MoS-ML [9,20].

Perturbations had a strong effect on variabilities, indicating that step irregularity is a specific characteristic of walking adaptability during perturbed walking [10,11,13,21]. Our results suggest that looking at the variability of parameters over a series of steps is a responsive measure of gait adaptations happening during perturbed walking. Importantly, it should be noted that in this method, the effect of the perturbations on the mean of the parameters could be smeared out, since it was measured over a series of steps and not over every single step after the perturbation. Despite limited responsiveness for measuring the effects on means, the presented approach of capturing the variability may represent a useful measure in future studies estimating fall risk in fall-prone populations. For instance, in a recent study, Punt et al. reported no difference between fallers and non-fallers ability to cope with perturbation when measuring mean of the parameters over every single step following the perturbation [28]. In their study, the effect of the perturbations on gait variability over series of steps (i.e. fluctuations) was not investigated, which might be helpful in providing additional information to discriminate between fallers and non-fallers. Our findings of high responsiveness of variability parameters are in agreement with Terry et al. who reported variabilities of CoM position and step width as the most sensitive parameters in response to continuous visual and mechanical perturbations toward ML-direction [13]. Also, in a recent study, Stokes et al. reported a more profound effect of continuous visual ML perturbations on variabilities of step width, step length, and trunk sway [11].

Significantly greater variability in response to ML perturbations indicates that to maintain stability, participants needed to exert greater control in response to ML perturbations [10,21,35]. The variability of SL was strongly affected by both ML and AP perturbations, whereas the effect of ML perturbations on the variability of SW was much greater than the effect of AP perturbations. MoS variability increased during all perturbed walking conditions. However, similar to variabilities of gait parameters, the variability of MoS was also greater for ML perturbations, as reported previously [12], reflecting the increased fluctuations in the placement of protective stepping after the onset of the perturbation in order to enhance stability [27]. Additionally, the variability of MoS-AP during the forward perturbation increased which was also reported by Young et al., demonstrating higher fluctuations of MoS-AP in the forward direction [12]. In the present study, gait instability was analyzed using an approach similar to that used by Lipsitz et al. [36] measuring heart rate variability and by Hausdorff et al. [37] measuring gait variability. The higher variability (i.e., more fluctuations) during and immediately after recovery stepping may be referred to as unsteadiness. In this sense, the variability of gait and stability parameters may be used as a marker of unsteadiness, instability, and fall risk. This should be further explored by applying this method in older adults and impaired population since not all variability is a mark of poor locomotor control. As in heart rate variability, some variability may reflect adaptability and be beneficial especially after an unexpected loss of balance. Indeed, the ability to adapt gait when negotiating unexpected hazards is crucial to maintain stability and avoid falling [38]. In the present study, the healthy young participants experienced no difficulty and no fall during perturbed walking. Thus, the high variability may show the ability of the young subjects to adapt the gait pattern which may be a healthy behavior to respond to unexpected perturbation. This initial work suggests that just as there is much to be gained by investigating gait and heart rate dynamics, above and beyond the study of the average heart rate and gait dynamics, similar investigations of step dynamics after an unexpected loss of balance may provide insight into postural stability and may also have clinical applications.

ML perturbations resulted in a deviation from the straight walking trajectory. Consequently, a lateral step or a crossing step was necessary to prevent sideward fall. Probably, increasing the step width causing increased MoS-ML which results in decreasing the risk of a sideward fall was prioritized above the stability in AP-direction. Therefore, participants in this study increased the variability of AP responses as well as ML responses to compensate for the higher risk of fall following the ML perturbations by taking wider and shorter steps. But AP perturbations resulted in an interruption of the forward progression. In this case, the risk of fall in backward and forward direction could decrease, respectively, by taking a backward or a fast and short forward step which resulted in the higher effect on the variability of AP responses than on ML responses. This observation suggests that presenting the ML perturbations affected stability in both ML- and AP-directions with a stronger effect in sideway fall than AP falls, and AP perturbations resulted in a stronger effect in the direction of the presented perturbation.

Backward perturbation reduced the distance between the anterior border of the BoS and the XCoM thus increased MoS-AP. It should be noted that increase in MoS-AP simultaneously might have the disadvantage increasing the risk of a backward loss of balance. Consequently, subjects took wider steps to recover stability. The increased step width during backward perturbation resulted in a greater MoS in ML-direction. However, the results of backward perturbation in this study should be interpreted with some cautions. Backward perturbations were presented by deceleration of the treadmill belt, which was accompanied by a sudden stop in the belt movement. Thus, gait cycles included in the backward perturbation consisted of gait cycles before and after the belt stop, and motion’s frames related to the stop of the belt were excluded from the analysis.

Extremes related to ‘high gait quality’ (HGQ) contain information about the best possible performance in the high-risk situation, whereas extremes related to ‘low gait quality’ (LGQ) contain information about responding to the risk which is related to the more demanding situations [29]. Therefore, together with the findings of this study, HGQ parameters are related to responses which show larger stride length (SL_P90_), shorter step width (SW_P10_), lower cadence (cadence_P10_), higher MoS-AP (MoS-AP_P90_), and lower MoS-ML (MoS-ML_P10_). While, LGQ parameters are expected to represent subject’s responses in the high-risk situations (i.e. during perturbations) which show shorter stride length (SL_P10_), larger step width (SW_P90_), higher cadence (cadence_P90_), lower MoS-AP (MoS-AP_P10_), and higher MoS-ML (MoS-ML_P90_).

HGQ parameters during perturbed walking exhibited no difference with that of normal walking. Thus, they showed no sensitivity to perturbations. As suggested by Rispens et al., perhaps the HGQ extremes are an accurate estimation of the individual’s capacities and do not capture the effect of perturbations [29]. Therefore, they showed the capacity and the best performance of young healthy adults in response to perturbations which exhibited no difference with normal walking.

Interestingly, the results of LGQ for all parameters were similar with the results of means and showed the same response pattern. However, the effect of LGQ of parameters was somewhat more significant and stronger compared to means. Thus, it seems that LGQ were more responsive and might be representative of the effect of unexpected perturbations.

There are some limitations in this study. First, due to technical limitations of the treadmill, all expected numbers of perturbations were not presented. Second, trials were not presented in a randomized order, therefore, the results of each condition could be influenced by learning of the previous condition. However, this fact does not interfere with the findings of this study since the main goal of this exploratory experiment was to find the effect of perturbations on spatiotemporal gait and dynamic stability parameters in order to evaluate the most sensitive measures which can better represent the effect of perturbations. Third, the data came from a fairly small sample of relatively healthy young adults. Thus there is a need to investigate larger sample sizes and explore older and "weaker" populations. Forth, there was no reflective markers attached to the treadmill. Consequently, the exact frame in which the perturbation was presented was undetectable. To address this limitation, all parameters were measured over a series of recovery steps and not over every single step after the perturbation. In this study, the extreme of the parameters may have captured the immediate effect of the perturbations on the parameters. Therefore, the present approach may potentially capture both the local effects (extremes) and the fluctuations over a series of steps (variability), although this needs to be validated in future studies. The detected information on extremes and variability of the parameters should be clinically validated as a fall risk assessment by applying this method on fall-prone populations. We acknowledge that the method of measurement over series of steps from a perturbation trial arose some limitations such as missing the subtleties that happen around the single steps following the perturbation. While the approach of analyzing a series of steps provided interesting information about the variability, it may smear out the effects of means. Therefore, the effect of the perturbations on the immediate steps after the perturbations should be investigated in future studies. In addition, the moment of the perturbation was adjusted to mid-stance of the left foot. However, there was a delay in triggering of the perturbations due to limitations in the setup of the treadmill device and since we could not detect the frame in which the perturbation was presented, the exact moment of the perturbations could not be determined. Thus, some cautions in interpreting the results should be taken into account, considering that depending on the moment of the perturbation within the gait cycle the response is different.

## Conclusions

The results show that the increase in cadence and step width, as well as the decrease in stride length, are strategies to increase MoS, and thus to decrease the probability of falling in the presence of perturbations. The present study also suggests that frontal plane fluctuations (ML variability) are more variable compared with AP variability. Thus, the variability of responses depends not only on the status of the individuals but also depends on the type and direction of the perturbation. The participants were more sensitive to ML perturbations than to AP perturbations which shows the importance of including ML perturbations in assessment protocols. Variabilities, as well as extremes of gait-related parameters, showed strong responses for measuring the effects of perturbations. Therefore, measuring variabilities and extremes of the parameters in addition to means can help to better understand balance control strategies and may be used as a marker of unsteadiness, instability, and fall risk. Further studies need to evaluate whether similar postural responses exist in older adults with different balance control abilities, such as between fallers and non-fallers. In this context, this study can be useful for designing advanced stability and gait evaluation and for introducing novel assessment protocols for estimating fall risk.

## Supporting information

S1 Data Data of the gait characteristics and dynamic stability. Parameters including SL, SW, cadence, MoS-ML, and MoS-AP were measured over each gait cycle during the time frames of interest in each walking condition.
(XLSX)
